# Protein and Metabolite Analysis Reveals Permanent Induction of Stress Defense and Cell Regeneration Processes in a Tobacco Cell Suspension Culture

**DOI:** 10.3390/ijms10073012

**Published:** 2009-07-06

**Authors:** Rico Lippmann, Stephanie Kaspar, Twan Rutten, Michael Melzer, Jochen Kumlehn, Andrea Matros, Hans-Peter Mock

**Affiliations:** Institute of Plant Genetics and Crop Plant Research (IPK), Corrensstraße 3, D-06 466, Gatersleben, Germany; E-Mails: lippmann@ipk-gatersleben.de (R.L.); kaspar@ipk-gatersleben.de (S.K.); rutten@ipk-gatersleben.de (T.R.); melzer@ipk-gatersleben.de (M.M.); kumlehn@ipk-gatersleben.de (J.K.); matros@ipk-gatersleben.de (A.M.)

**Keywords:** apoplast, suspension culture, tobacco, extracellular proteins, secreted putrescine

## Abstract

The secretome of a tobacco cell suspension culture was investigated by a combined proteomic and metabolomic approach. Protein analysis from 2-DE gels led to identification of 32 out of 60 spots from culture medium. Identified proteins were mainly involved in stress defence and cell regeneration processes. Among them three putative new isoforms, e.g. for chitinase, peroxidase and β-1,4-xylosidase were identified, not yet present in available protein databases for the genus *Nicotiana*. GC-MS analysis of time course experiments revealed significant changes for metabolites involved in energy transport, signalling and cell development. Among them, the most significant increase was found for putrescine in the medium of cultures entering the exponential phase. Results showed strong abundance of stress associated proteins and metabolites in the absence of elicitors or additional stress treatments.

## 1. Introduction

During recent decades plant tissue cultures have been used extensively to study cell growth and metabolism. Like their wild relatives plant cell cultures have been shown to also synthesize a wide variety of secondary metabolites, often with highly complex structures and of pharmaceutical importance. As chemical synthesis of relevant secondary compounds is often economically not feasible, isolation from plants is still the method of choice. Although biotechnological production using plant tissue cultures has been discussed as an alternative, its commercial use is still limited due to the lack of knowledge about biosynthetic pathways and their regulation [1]. On the other hand, synthesis of secondary compounds *in vivo* is often related to recognition of abiotic or biotic stress conditions, which also have a big impact on yield and quality of crop plants used for feeding animals and human nutrition. To study biosynthetic pathways plant tissue cultures are often used as they are easy to cultivate, and manipulation of secondary metabolite production in the non-differentiated cells can be done by treatment with elicitors (e.g. methyl jasmonate, salicylic acid and chitosan) as well as by application of abiotic stress such as UV-light [2]. Much work has been done in the last decades with regard to investigation of specific pathways such as those of phenylpropanoid, alkaloid, and terpenoid biosynthesis [3–5]. Taking advantage of recent developments for comprehensive analysis of complex biological systems (e.g. proteomics, transcriptomics, and metabolomics techniques) more detailed analyses of molecular and cellular changes in plant cell cultures have been performed. As an example recent studies of the effects of biotic and abiotic elicitors on metabolism in the model legume *Medicago truncatula* revealed strong correlations between changes in the primary metabolite pool and changes to secondary metabolism at the metabolite level [6] as well as on the transcript level [7]. A number of other studies combining the approaches named above have consistently provided new insights into metabolic regulation of plant stress responses at the cellular level [8,9]. In addition, comprehensive mapping of protein pattern as shown for a suspension culture of *Medicago truncatula* [10] should provide a useful tool for future comparative protein analyses and for functional genomic studies.

However, most investigations have been focused on biochemical changes in the cells. Plants secrete in addition a variety of proteins and metabolites into the extracellular space (apoplast) or bind them to the cell wall [11–13]. In fact, apoplast function is crucial for plant life and includes: (I) growth regulation, (II) tissue structure, (III) defence against abiotic and biotic stress factors, (IV) transport, (V) osmotic homeostasis, (VI) cell adhesion, (VII) and gas exchange [14]. In early reports the capability of cultured cells of *Nicotiana tabacum* to secrete vacuolar proteins like *α*-mannosidase, class I chitinase and class I *β*-1,3-glucanase has been shown [15]. Cell suspension cultures were also used in many studies to investigate stress and defence reactions in the apoplast. Thus, a secreted lipase was identified to be directly associated with ethylene signalling in response to infection of *A. thaliana* suspension culture cells with the necrotrophic fungus *Alternaria brassicicola* [16]. On a metabolic level studies have identified many secondary metabolites secreted into culture media [17,18]. Moreover, a number of proteins were identified from the apoplastic fraction of an *Arabidopsis* cell culture without stress treatment, mainly related to cell wall regeneration, but also to stress response [19]. All together, this indicates a permanent induction of cell division processes in coordination with stress response mechanisms. Here we have investigated the extracellular components from a tobacco cell suspension culture without further stress treatment and found more than 60 proteins present in the medium. Furthermore, in addition to well known defence related and secreted secondary metabolites like scopolin, other metabolites related to stress and cell development were identified in the medium. Most of the secreted components can be related to defence or stress response and cell wall modification.

## 2. Results

### 2.1. Growth and Viability

**Figure 1.**
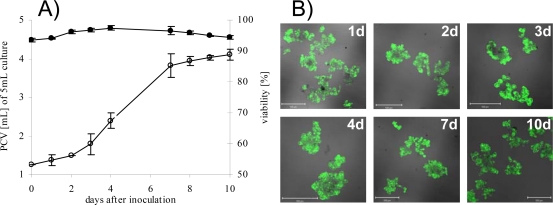
Growth curve and viability of S2LS3 cell culture. A) Growth rate as packed cell volume (PCV) out of 5 mL (n=4, open circle). The viability of cell culture was counted after labelling with the vitality dye fluorescine diacetate (FDA) as percentage of living cells (black circle). Error bars represent the standard deviation of mean values. Over 500 cells were counted for calculation (n=2) B) Fluorescence images of different time points. Cell culture was stained with FDA to monitor cell viability and cells emitting green fluorescence were counted as vital. Fluorescence was visualised in a CLSM using a 488 nm laser line in combination with a 505–530 nm band pass filter. The scale bars represent 500 μm on all images.

S2LS3 cells were cultivated as described in the Experimental Section. For initial characterization growth rate and viability of cells were evaluated for 1, 2, 3, 4, 7, 8, 9 and 10 days of cultivation time. The growth of S2LS3 cell suspension culture showed a standard sigmoid curve with a lag-phase (0–2d), log-phase (3–7d) and a stationary phase (8–10d) (Figure 1A, open circle). The following typical subculture periods were 7 days. To estimate the viability, cells were stained with fluoresceine diacetate (FDA). No significant changes in cell vitality were observed over the whole cultivation time and on every time point at least 93 % of collected cells were vital (Figure 1A, black circle, and Figure 1B). This finding indicated that the culture medium used for analyses was not or only marginally contaminated with cell fragments or intracellular compounds. For protein analysis, samples were harvested at day 7, the end of log-phase. Samples for metabolite analysis were taken at different time points to estimate kinetic trends of accumulation.

### 2.2. Identification of Secretory Proteins

**Figure 2.**
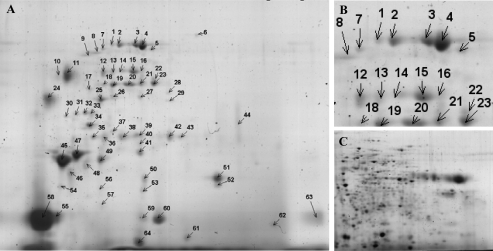
Protein separation by two-dimensional gel electrophoresis (2-DE). A) 2-DE analysis of total secreted proteins of S2LS3 cells after 7 days of cultivation. Separation was performed in 1^st^ dimension at pH 3-10 and in 2^nd^ dimension with 11.25 % acrylamide. After staining with CBB over 60 spots were detectable. B) Enlarged view of marked region from 2-D gel of secreted proteins from S2LS3 cells. C) Representative 2-D gel of intracellular proteins from cell culture for comparison with protein intensities of secreted proteins.

**Figure 3.**
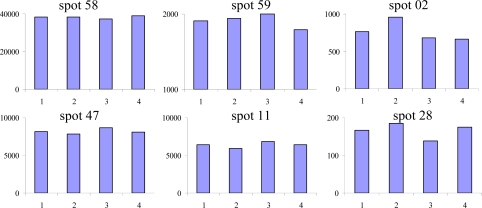
Comparison of spot intensities from biological replicates. Selected low (#58 & 47), middle (#59 & 11) and high (#02 & 28) abundant proteins from four biological replicates calculated with Progenesis SameSpots software were shown. Intensities are presented as normalized spot volume and showed no significant changes between individual replicates.

Preliminary experiments for analysis of extracellular proteins showed increasing accumulation of proteins in the cell culture medium during the log phase, and proteolytic degradation becoming apparent only at day 10 of cultivation. The concentration of accumulated proteins varies during cultivation time, showing highest protein concentrations in the medium at day 7. Thus, we have chosen to use the medium from the end of the log-phase at day 7 of cultivation for analysis of the secreted proteins to get the highest recovery of secreted proteins. The patterns of proteins secreted by the cells were analyzed in four independent experiments. After 2-DE separation and staining with colloidal CBB, more than 60 spots were visualized in all replicates. A characteristic 2-DE image is shown in Figure 2A. Reproducibility of individual 2-DE images of all experiments was evaluated using image analysis software. Comparison of spot intensities showed good reproducibility between the 4 biological replicates (Figure 3). From all spots pieces of similar size were excised, proteins were digested with trypsin and digests analyzed by nanoLC ESI-QTOF-MS/MS. In total, 32 out of 64 Spots could be identified. Table 1 presents the protein name, accession number, biochemical properties, as well as peptides leading to the respective identification.

**Figure 4.**
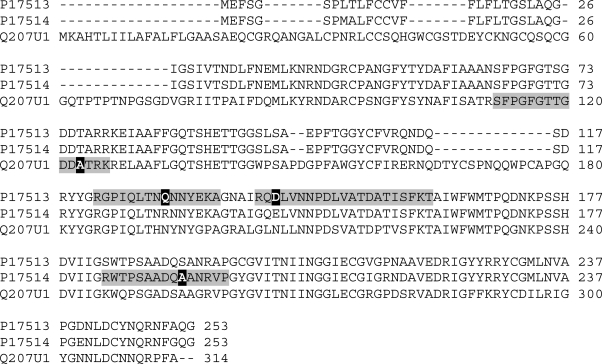
Alignment of amino acid sequences from chitinase isoforms. Two isoforms of endochitinase P (P17513) and Q (P17514) from *N. tabacum* and one chitinase (Q207U1) from *M. charantia were aligned.* Identified peptides for each chitinase were marked in grey, and in black amino acids are signed which are unique for isoforms. Alignment was performed with CLUSTAL 2.0.10 multiple sequence alignment from UniProt.

Most of the proteins present in the secretome of S2LS3 cells were identified as β-xylosidase. Peptides belonging to this protein were found in several series of protein spots, e.g. spot #1 to 4, spot #12, 14, 15, and spot #18, 20, 21, 22, 23 (refer also Figure 2B). In addition, some single protein spots from various regions of the 2-D gels were identified as β-xylosidase; e.g. spot #27, 41, and 52 (refer also Figure 2A). Although β-xylosidase was identified in all the protein spots mentioned above, identification relied on various protein ID’s from several organisms, e.g. *Populus tremula*, *Solanum lycopersicum*, and *Arabidopsis thaliana* (refer Table 1). Among these a database search of corresponding peptides also led to the identification of one EST-clone from *Nicotiana* (e.g. TC13056). Analysis of tryptic peptides from spot #45, 46, and 47 lead to identification of an acidic endochitinase from *Nicotiana tabacum*. Thereby, peptides for acidic endochitinase isoform P (P17513) were found in spots #45, 46, and 47, whereas peptides for acidic endochitinase isoform Q (P17514) were exclusively found in spot #47. Moreover, an additional peptide leading to identification of a chitinase from *Momordica charantia* was found for spot #47 (refer Table 1). This protein only showed a homology of 54.2 % to P17513 and 55.1 % to P17514. In Figure 4 alignment of protein sequences from acidic endochitinase isoform P (P17513), isoform Q (P17514) and the chitinase from *M. charatnia* is shown. Peptides which led to identification of the respective protein are indicated in grey, and black marked amino acids show the difference to the other isoforms. Peptides diagnostic for each protein could be found.

Database search identified spots #55 and 58 as pathogenesis related protein (*N. tabacum*). However, from the identified peptide it was not possible to distinguish between the three isoforms of pathogenesis related protein, 1A (P08299), 1B (P07053), and 1C (P09042); refer also to Table 1.

Furthermore, in the set of secreted proteins subtilisin like protease isoforms from *N. tabacum*, A9XG40 and A9XG41 were found in spots #6 and 57. Several peptides have been detected for both spots leading to the same identification, however only in spot 57 could a unique sequence for both isoforms be identified.

In addition, a number of defence-related proteins and proteins involved oxidative stress metabolism were identified, e.g. spot #11 as peroxidase and spot #24 as lignin forming anionic peroxidase, among others.

Among the identified proteins, several were identified in multiple spots on the 2-D gels, e.g. spots #25 and 35 were annotated as α-galactosidase, spots #34 and 57 as glucan-*endo*-1,3-β-glucosidase acidic isoform PR Q, spots #36 and 49 as biotic cell death associated protein, and spots #37 and 38 as xyloglucan endotransglucosylase, respectively (see also Table 1).

In principle, secreted proteins could also accumulate in a culture medium as a result of cell damage. However, our results clearly indicate that the protein patterns observed resulted from secretion of proteins. Firstly, cell viability was very high and only few cells showed cell death also after seven days (refer also to Figure 1). Secondly, protein patterns of intracellular proteins were clearly different from the secretome. A characteristic 2-DE image from an intracellular protein fraction of S2LS3 cells is shown in Figure 2C. Comparative image analysis of spot patterns from secreted with intracellular proteins showed little overlap. Moreover, most of the identified proteins were predicted to have a signal peptide and/or to be secreted (cf. Table 1). For calculations the SignalP 3.0 program (http://www.cbs.dtu.dk/services/SignalP) was used with the following settings: as organism group eukaryotes were selected and the prediction method was the hidden Markov model. Predicted signal peptide sequences have been accepted when showing a higher probability than 0.55.

**Figure 5.**
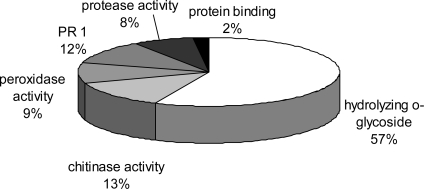
Proteins clustered to their functional activity. Functional activity and processes were clustered according to GO annotation (UniProt protein database) of biological process.

For annotation of the identified proteins according to their putative function, the criteria of UniProt protein database (GO annotation) were followed (Table 1). In summary, all identified proteins were either related to stress responses, defence reactions or cell wall metabolism. Thereby, some proteins described to be involved in cell wall metabolism were also annotated as being stress or defence response related in the database. These proteins belong to the cluster of pathogenesis related (PR) or pathogenesis related like (PRL) proteins containing members from different protein subfamilies [20]. In Figure 5 a pie chart is shown, representing the grouping of identified proteins due to their molecular or biochemical function as taken from GO annotation. Most proteins were found to exhibit glycosidase activity, followed by proteins with chitinase, peroxidise, and protease activity. In addition a group of PR1 proteins was annotated with still unknown biochemical function.

### 2.3. Secreted Metabolites

**Figure 6.**
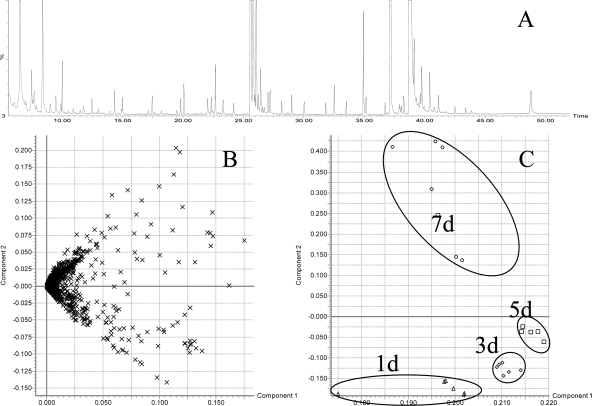
GC-MS analysis of extracellular metabolite profiles from S2LS3 cell culture medium after derivatisation. Exact mass retention time pairs (EMRT) were used for principle component analysis (PCA). A) Representative total ion current (TIC) chromatogram at day 7. The y-axis was set to 25% of the base peak. B) Loading Plot from PCA. C) Score Plot calculated from the Loading Plot with component 1 and 2. Samples belonging to the same cultivation time (1d, 3d, 5d, and 7d, n = 6) were marked with black circles.

Secreted metabolites were analyzed by a GC-MS approach, which is a powerful tool, as a wide range of compounds can be measured within a single chromatographic run [21]. Metabolite profiles from different time points (day 1, 3, 5 and 7) were analyzed from six biological replicates as described and acquired data were used for principle component analysis (PCA). A typical total ion current chromatogram (TIC) is shown in Figure 6A. Within the extracted time window of 6 to 37 min, 2,481 exact mass retention time pairs were used for PCA with the parameters described in the Experimental Section. From the raw data a Loading Plot was calculated by MarkerLynx software (Figure 6B) and transferred to the reduced Score Plot within the PCA analysis (Figure 6C). The calculated clusters showed a strong correlation with the different time points. After extraction of the responsible exact mass retention time pairs (EMRTs) saturated ions were excluded from the data set because of their high variance. In fact, these EMRTs belong to the medium components, such as sucrose, the monosaccharides glucose and fructose derived from sucrose and inositol. The intensities of these most abundant signals decreased over time consistent with the consumption of sugars by the growing cell culture (data not shown). For the annotation of metabolites, the NIST 05 library was utilized. Whenever available reference compounds were also analyzed to check the retention time correlation and fragment spectra. Metabolites showing altered profiles during cultivation were identified as galactose, benzoic acid, inositol, and three other sugar furanoside derivatives which were not further identified yet. Among them, galactose and putrescine increased with time, whereas benzoic acid was detected in a low density over the whole cultivation time. Interestingly, myo-inositol an important factor for cell growth and supplemented to medium was undetectable after day 3. Intensities and retention time of these metabolites are shown in the supplementary data (Table S1).

**Figure 7.**
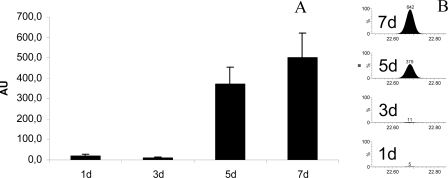
Relative quantification of the secreted metabolite putrescine. A) Relative concentration of putrescine over the cultivation time (n = 5). The area of the typical fragment mass of putrescine *m/z* = 174.1 were used for elucidation. B) Extracted mass of *m/z* =174.1 showing the different peak areas of putrescine fragment as an example from one replicate.

Another fragment which showed a strong increase over the cultivation time was annotated as the diamine, putrescine. In labelling experiments with ^15^N, the production of putrescine from cell culture was proven and a shift from ^14^N to ^15^N isotope fragments was detected (supplementary data, Figure S1). For the relative quantification of secreted metabolites the peak areas of a typical unique mass of putrescine (m/z=174.1) were used. Results, shown in Figure 7, clearly indicate a strong increase of secreted putrescine after three days of cultivation.

However, for absolute quantification of putrescine targeted extraction and analysis with HPLC is more suitable than GC-MS [22–24]. Thus, targeted extraction of polyamines with perchloric acid, a derivatisation with dansyl chloride and subsequent analysis with HPLC coupled to fluorescence detection was performed. A representative chromatogram is shown in the supplementary data (Figure S2). Advantages were a higher sensitivity and a better reproducibility of signal intensities from independent samples. Furthermore, samples were harvested on five time points (1d, 3d, 4d, 5d, and 7d) to also investigate the dramatic increase between day three and five, as seen in the GC-MS analysis. As expected the kinetic trends showed similar results like GC-MS analyses. For absolute quantification a calibration curve with authentic putrescine was used. The concentration of putrescine showed a slow increase within the first three days from 4 nmol·mL^−1^ to 15 nmol·mL^−1^. On day four, putrescine increased 20 fold to 84 nmol·mL^−1^ as compared with day one. After this dramatic rise only a slight further increase (to 115 nmol·mL^−1^) was observed, indicating that biosynthesis and/or secretion occurred prominently at the onset of the exponential growth phase.

## 3. Experimental Section

All chemicals were of analytical grade or higher and were obtained from Sigma-Aldrich (Munich, Germany), Roth (Karlsruhe, Germany) or Merck (Darmstadt, Germany).

### 3.1. Growth and Viability of Cell Culture

The cell suspension culture was derived from a root callus of *N. tabacum* L. cv Havana SR1 [25]. Cells were grown in Linsmaier-Skoog medium [26] LS-3 supplemented with 30 g·L^−1^ sucrose, 2 mg·L^−1^ of the auxin α-naphtalene acetic acid (NAA) and 0.3 mg·L^−1^ of the cytokinin kinetin. Erlenmeyer flasks (100 mL) with 20 mL of culture medium for end point experiments, and Erlenmeyer flasks (250 mL) containing 70 mL of culture medium for time course experiments were used. Subcultures were started by inoculation with 4 mL of 7 days old stock suspensions and cultivated under continuous shaking at 110 rpm at 25 °C in the dark. The growth was measured as packed cell volume of 5 mL suspension culture. Centrifugation at 100 × g for 5 min at 25 C, the volume of sedimented cells was measured using a graduated centrifuge tube. Viability of cell culture was estimated daily by staining with fluorescine diacetate [27,28].

### 3.2. Extraction and Purification of Proteins

Culture medium of cell suspension was obtained by three filtration steps. First, cells were removed through Nylon mesh with pore diameter of 100 μm, followed by filtration through Nylon mesh of 25 μm pore size. After that, filtration through cellulose acetate (0.45 μm, Sartorius) was performed to remove cell debris. The filtrate was frozen in liquid nitrogen, and freeze dried or directly used for metabolite analysis. The dried material was resolved in one tenth of the original volume. Ten μL of dissolved medium were used for protein quantification with Bradford assay [29] using bovine serum albumin as a standard. Finally an aliquot of the protein extract was precipitated with methanol/chloroform as described by Wessel and Flügge [30] to concentrate proteins prior to subsequent 2D gel electrophoresis. The pellet was dried under nitrogen at 30 C.

### 3.3. Two Dimensional Gel Electrophoresis (2-DE)

The pellet from protein extraction was dissolved in lysis buffer containing 8 M urea, 2% (w/v) CHAPS, 20 mM DTT, 0.5% IPG buffer as described by Amme *et al*. [31]. Insoluble material was removed by centrifugation (15 min, room temperature, 20,000 × g). Additional supernatant was clarified with 0.45 μm filter units (ULTRAFREE-MC; Millipore, Eschborn, Germany). The protein concentration was determined using the 2-D Quant Kit (GE Healthcare) following the manufacturer’s instructions. Fifty μg of protein was loaded onto IPG strips (7 cm, pH 3–10) by rehydration on a IPGPhor 2 unit (GE Healthcare) and IEF was carried out at following parameters: active rehydration step of 14 hrs (50 V) at 20 °C, then 60 min gradient to 250 V, 60 min gradient to 500 V, 60 min gradient to 4,000 V and 5.30 hrs 4,000 V with a total of about 25 kVh. After isoelectric focusing, strips were equilibrated and separated on a SDS polyacrylamid gel (SDS-PAGE, 11.25% acrylamide) in a Hoefer S600 apparatus (GE Healthcare).

### 3.4. Digestion and Identification of Proteins with LC-ESI-QTOF-MS/MS Analysis

Pieces with a diameter of 1.5 mm were picked from the middle of the spots and washed for 30 min with 400 μL of 10 mM ammonium bicarbonate/50% acetonitrile (CAN). After removing the washing solution, gel pieces were dried under vacuum. For protein digestion, 10 μL of trypsin [Promega Sequencing grade, 10 ng·mL^−1^ in 5 mM ammonium bicarbonate/5 % ACN (w/v)] were added and incubated for 5 hrs at 37°C. Reaction was stopped by adding 2 μL of 1% TFA. For identification 2 or 3 μL of tryptic digest was subjected to nanoscale reversed-phase LC analysis on a nanoAcquity UPLC system (Waters Corporation, Eschborn, Germany). A 20 mm × 180 μm Symmetry 5 μm column was used for concentration and desalting of the digested samples. The subsequent peptide elution was performed on a 100 mm × 100 μm BEH130 C18 1.7 μm column (Waters Corporation) with an acetonitrile gradient increasing from 3% to 40% in 30 min. The efflux from analytical column was directed to the NanoLockSpray source of a Q/Tof Premier hybrid orthogonal accelerated time-of-flight mass spectrometer (Waters). MS and MS/MS data were acquired in continuum mode using MassLynx 4.0 software (Waters Corporation). Data processing and database searching of de novo sequences against protein indices for *Viridiplantae* of the SwissProt and TrEMBL databases, as well as against a combined EST database of TIGR EST sequences from *Solanaceous* species was done using ProteinLynx GlobalSERVER v2.3 software (Waters Corporation). A 10 ppm peptide, 0.1 Da fragment tolerance, one missed cleavage and variable oxidation (Met) and propionamide modification (Cys) were used as the search parameters.

### 3.5. Derivatisation of Metabolites from Culture for GC-MS Analysis

Ten μL of filtered cell culture medium were dried *in vacuo* for 2 hrs at 35°C. The dried residue was redissolved and derivatized in a first step with 40 μL of methoxyamine hydrochloride (20 mg·mL^−1^ in pyridine) for 2 hrs at 37 °C to convert keto- and aldehyde groups. In a second step, 80 μL MSTFA was added to each sample and incubated for 30 min at 37°C. One μL of sample were injected with splitless injection method using hot-needle technique. The GC-MS system consisted of a gas chromatograph A7890 (Agilent, Böblingen, Germany), an auto sampler Twister XXL (GERSTEL, Mülheim an der Ruhr, Germany) and a TOF mass spectrometer GCT Premier (Waters Corporation). Separation was performed on a 30 m DB5ms column (Phenomenex, Aschaffenburg, Germany) with 0.25 mm inner diameter and 0.25 μm film thickness including a 5 m guard column. Injection temperature was 240°C. Temperature program for GC separation was: 3 min 80°C isothermal followed by a ramp of 5°C min^−1^ to 310 C for 5 min. The system was equilibrated for 3 min prior each injection. MS data were recorded with Mass Lynx 4.1 (Waters Corporation) with 10 spectra s^−1^ in a range of 50–650 m/z in centroid mode and using the dynamic range enhancement (DRE) modus.

Metabolites were identified automatically with internal software ChromaLynx using the NIST 5 library and interesting components were validated manually by comparison with reference spectra and with retention time of individual standards. For principle component analysis (PCA) MarkerLynx (Waters Corporation) was utilized with following settings: 20 masses per retention time were isolated at a threshold at 5% of base peak intensity. The retention time window was set between 6.5 and 37 min, and mass window was set between 100 and 850 with a mass tolerance at 0.05 Da Pareto algorithm was used for visualization of Loading and Score Plot.

### 3.6. HPLC Analysis of Polyamines

The culture medium was centrifuged through 0.45 μm filtration units (ULTRAFREE-MC; Millipore, Eschborn, Germany) to remove potential cell debris and stored at −20°C until further processing. Derivatisation was performed following a modified protocol of Silveira [23]. A 20 μL aliquot of medium was mixed with the equal volume of 5% (v/v) perchloric acid and added to 100 μL 18.5 mM dansyl chloride dissolved in acetone, 10 μL 0.05 mM diaminoheptane as internal standard and 50 μL saturated sodium carbonate. After incubation at 70°C for 50 min in the dark, 25 μL of 0.87 M proline was added to remove excess dansyl chloride by conversion to dansyl proline. Polyamines were extracted after 30 min with 200 μL toluene in a two phase partitioning step. To avoid carryover from the aqueous phase only 150 μL of toluene was transferred to a new reaction tube and dried under nitrogen. The residue was redissolved in 150 μL acetonitrile. Metabolite analysis was carried out on 10 μL of sample using an Alliance HPLC separations module 2690 (Waters Corporation). Fluorescence signals were acquired with a FP-920 detector (Jasco, Gotha, Germany) at 340 nm (excitation) and 510 nm (emission) with a signal gain of 100. A calibration curve from a dilution series of putrescine was applied for quantification.

## 4. Discussion

### 4.1. Secreted Proteins Related to Cell Development as well as Stress Defence

The advantage of analyzing extracellular compounds from cell culture media is that little or no contamination from intracellular fragments is found compared to other studies using xylem sap or apoplast in which also typical intracellular proteins have been detected [13,32]. In our study no typical intracellular proteins were found and analysis of proteins for signal peptides revealed a classical secretory pathway for most of identified proteins. The comparison of intra- and extracellular protein pattern showed only a minor overlap of spots (spots #58 and 59), but with a significant higher abundance of proteins in the extracellular fraction. These results and the high cell viability, indicate that there is neglectable contamination of the culture medium with intracellular proteins. In accordance, enzyme tests from earlier studies with the same suspension culture showed only minor enzyme activity of the intracellular malate dehydrogenase [15]. Most gene products found in the medium were cell wall modification proteins. Among this, the protein 1,4-β-xylosidase was dominating and found in multiple spots. As already mentioned in the Results section, the identification relied on different ID’s from several organisms including identification of one EST-clone from *Nicotiana* (TC13056), which indicates related polypeptides for tobacco not included in the protein databases so far. Furthermore, different α-galactosidase polypeptides were identified which are supposed to be responsible for cell wall modification like elongation [33].

The multiple spot abundance of 1,4-β-xylosidase, and others, could be regulated by differential gene expression of isoforms and/or posttranslational modifications, such as glycosylation and phosphorylation and has to be determined. Glycosylation is well known from literature for extracellular proteins, which showed a high rate in most cases especially for xylosidases and galactosidases [19]. Phosphorylation on the other hand is a key regulator mechanism for many signal transduction pathways, e.g. important for the induction of defence response in elicitor induced *Zea Mays* extracellular matrix proteome [34]. The same modifications could also play a role in non treated cell cultures in stress response or in senescence [35]. Besides, the abundance of a subtilisin like protease from *N. tabacum* suggested that further protein degradation is existent to decrease the abundance, and those the activity of these enzymes.

Other polypeptides detected in culture medium were related to stress defence or pathogen attack. The two acidic endochitinases P & Q identified in the medium belong to class III chitinases. Both have been known for several years [36] and are classified as PR-3 family proteins. A third chitinase was identified related to a sequence entry from *M. charantia*. This protein was identified by a unique peptide with a sequence not found in the other two isoforms. This result indicated a further putative isoform from tobacco, without an entry in the tobacco sequence database. Chitinases can be expressed constitutively in healthy plants. The class III chitinase transcripts for example are constitutively present in vascular bundles or guard cells of *Cucumis sativus* and *Arabidopsis thaliana* [37], and additionally switched on by infection with a compatible pathogen in tissues around necrotic lesions [37]. Moreover, their regulation seems to be also controlled by hormonal feedback control, as the presence of auxin and cytokinin in *Nicotinana* sp. represses the class I chitinase genes but does not repress class III genes [38]. Chitinases were reported to have function in cell development and growth, and participate in the cell elongation process [39], as well as in somatic embryogenesis, in line with the complex regulatory pattern observed [40]. Hence, a distinct function of chitinases in our suspension culture system can not be attributed so far. The high abundance of these enzymes however is consistent with wounding and stress responses in the suspension culture but also with a high growth rate of the cell culture.

All the identified peroxidase proteins belong to the class III peroxidases [41] (http://peroxidase.isb-sib.ch). For spot #11 an EST-clone from *Nicotiana* showed a corresponding peptide sequence, but the protein is not found in a protein database suggesting a novel peroxidase isoform in tobacco which needs to be further investigated. However, peroxidases found in the medium are already annotated to numerous functions e.g. to remove H_2_O_2_, biosynthesis and degradation of lignin, suberization, auxin catabolism, as well as response to environmental stresses such as wounding, pathogen attack and oxidative stress. All together, the function of identified peroxidases could be dependent on specific isoforms. The most abundant spots #55, 58 out of 2-DE were identified as pathogenesis related 1 (PR-1) protein. Although the function of this protein is still unknown, the strong abundance is a clear indication for stress response.

Summarized our results from protein analysis of S2LS3 cell suspension culture medium revealed a strong stress activated response, although no elicitor treatment was used. All identified proteins belong to pathogenesis related protein families closely involved in the so called systemic acquired resistance mechanism. Many of them also play an important role in cell wall formation and restructuring. Both processes are linked to each other, because after wounding, cell wall modification is initialized. The stimulus for the observed protein patterns related to wounding could be the constant shaking of the cell culture, necessary to sustain oxygen supply.

### 4.2. Secreted Metabolites

Secreted metabolites found in the apoplast or suspension culture medium fulfil different functions. They play an important role in energy transport (e.g. sugars), signalling, cell development and/or stress defence mechanisms. Many primary and secondary metabolites, which show a stress induction in plant cell cultures, were identified over the last decades mainly during investigation of biosynthetic pathway or stress defence networks [42]. In this study we analyzed medium of tobacco suspension cultures in an un-targeted way without initial concentration of specific classes of molecules. As expected, the concentration of supplemented sucrose as well as of its constituents glucose and fructose decreased, being the carbon source for cell growth. Interestingly both monosaccharides were already present in culture medium before inoculation, as measured by GC-MS and confirmed with an enzymatic test (data not shown), which indicates degradation of sucrose during sterilizing the media by autoclaving. However, the amount of other sugar compounds like galactose was found to increase with time. This confirms the results from protein analysis in which glycosidases were identified. The sugars found in medium could be products from their enzymatic activity.

Furthermore, inositol as another constituent of the medium was only found to day 3 after inoculation. In eukaryotes, phosphoinositides are essential metabolites as well as effective messengers that regulate and coordinate cell growth. In fact, they have been shown to induce Ca^2+^ oscillations, to affect membrane biogenesis, cytoskeletal structure, ion transport, and RNA synthesis and transport. Due to their influence in so many cellular processes temporal shifts in phospho-inositol metabolism are known to occur with growth and development [43].

A remarkable increase during cultivation time was observed for the polyamine putrescine. We confirmed the GC-MS analyses in a targeted approach. Polyamines (PAs) are polycations ubiquitous in prokaryotic and eukaryotic cells and are essential for cell growth, proliferation and differentiation [44]. It was also referred that polyamines play an important role in abiotic or biotic stress response [45,46] and are involved in the hypersensitive reaction [47]. Although the exact mechanisms of many of these processes are unclear, it is well known that PA binds to negatively charged molecules like nucleic acids, acidic phospholipids or proteins [48]. Polyamines are a source for hydrogen peroxide as well [47,49]. The involvement into many cellular processes makes it difficult to attribute the excreted putrescine to a specific function. Lignification of cell wall needs a radical-radical coupling and precursors are formed from peroxidase or laccase of which the latter enzyme needs oxygen [50]. When cells reach a certain density their oxygen supply becomes limiting [51]. This indicates the demand of peroxidase activities for lignification and as already mentioned peroxidases need hydrogen peroxide for their enzymatic activity. How H_2_O_2_ is generated in cell walls is still a matter of debate. However, in *Arabidopsis* tracheary elements, an amine oxidase that produces H_2_O_2_ by oxidizing putrescine has been co-localized with lignin staining and peroxidase activity [30]. In our study, putrescine showed a strong correlation with cell culture growth, indicative to its connection with cell division and proliferation wherefore cell wall biosynthesis and lignification is needed. On the other hand the suspension culture is constitutively stressed as discussed above. As already mentioned, putrescine is a major source for H_2_O_2_ involved in cell death and early stress defence or rather hypersensitive reaction (HR) [49]. Therefore, the H_2_O_2_ built out of putrescine could also provoke additional stress to the tobacco cell culture.

## 5. Conclusions

Our results clearly demonstrate that also non stress induced suspension cultures excrete a high number of metabolites and proteins which concertedly affect stress defence mechanisms and cell structure modifications. Among identified proteins β-1,4-xylosidase was found in many spots indicating high level of posttranslational modification and protein degradation in cell culture media. Furthermore, putative new isoforms are predicted from peptide sequences of mass spectrometry analysis for chitinase, peroxidase and β-1,4-xylosidase, not described in tobacco yet. Potential functional differences for various isoforms of those proteins in the apoplast need to be evaluated in further studies. On metabolite level, a dominate putrescine accumulation in the medium was detected after 3 days which further increased with time in correlation with cell culture growth. These results suggest that, putrescine may be a critical component for cell division and proliferation. Thus, we could show that S2LS3 tobacco cell suspension culture is an excellent model system to investigate extracellular proteins and metabolites related to stress defence and cell development.

## 

Figure S1. A shift from the ^14^N to ^15^N isotope fragment of putrescine. A) The isotope shift after 14d of cultivation with ^15^N labelled culture medium. B) Natural isotope pattern of the putrescine fragment.

Figure S2. Representative HPLC chromatogram of polyamine analysis from 7d old culture media. Polyamines were measured by fluorescence detection at 340 nm (excitation) and 510 nm (emission). The identification of polyamines was performed with authentic standards.

**Table S1.** Putative identity of compounds detected in the medium of S2LS3 culture. Metabolites were identified by matching their spectra to NIST library and the Golm metabolomics database. Retention time (RT), retention index (RI) calculated with alkanes; the match values from Nist, and the intensities (peak area) of a compound-specific molecular fragment (specific ion, *m/z*) at different time points are shown. Values for peak area are given Not identified values were signed with n.i. and intensities under the detection limit signed with n.d.

**Identity**	**Specific ion (m/z)**	**RT [min]**	**RI**	**RI Golm DB**	**Nist Matc h**	**Rev. Nist Match**	**Peak area 1d ± STD**	**Peak area 3d ± STD**	**Peak area 5d ± STD**	**Peak area 7d ± STD**
**Lactic acid 2TMS**	190,99	6,58	1010	1051	883	884	492±124	606±218	726±188	667±66
**Benzoic acid TMS**	105,03	11,03	1244	1253	789	851	n.d	12±2	11±2	10±1
**Galactose methoxyamine 5TMS**	117,07	22,34	1714	1721	723	744	7±3	48±11	124±22	131±14
**Putrescine 4TMS**	174,11	22,68	1729	1745	894	895	20±9	9±2	369±80	492±115
**Unknown sugar 1**	217,07	23,31	1758	n.i.	n.i.	n.i.	246±77	272±73	191±38	93±10
**Fructose methoxyamine 5TMS**	364,00	25,62	1870	1865	873	874	1200±175	1440±249	1306±210	410±84
**Fructose methoxyamine 5TMS 2**	364,18	25,82	1880	1774	878	880	959±104	1142±200	286±30	360±70
**Glucose methoxyamine 5TMS**	364,18	26,07	1893	1888	908	923	455±65	286±30	151±9	19±3
**Glucose methoxyamine 5TMS 2**	364,18	26,39	1908	1910	828	918	11±1	10±2	2±0.5	n.d.
**Unknown sugar 2**	205,10	26,57	1917	n.i.	n.i.	n.i.	137±46	120±33	41±11	25±3
**Unknown sugar 3**	147,10	26,72	1924	n.i.	n.i.	n.i.	n.d	n.d	n.d	14±3
**Inositol 6TMS**	317,97	29,69	2082	2091	914	944	1380±260	4±1	n.d.	n.d.
**Sucrose methoxyamine 8TMS**	361,15	38,66	2636	2640	760	767	saturated	saturated	saturated	saturated

## Figures and Tables

**Figure 8.**
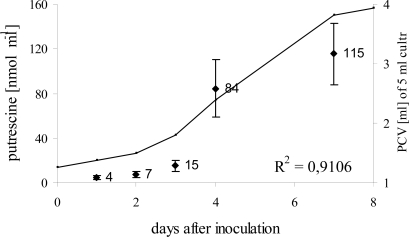
Dynamics of extracellular putrescine in the S2LS3 cell culture. The concentration of putrescine is plotted as black points, n = 5. Concentrations were calculated using a standard calibration curve. The cell growth is plotted as solid line. The correlation coefficient between both parameters is 0.91.

**Table 1.** Identification of secreted proteins from 2-DE analysis of culture medium. Identified proteins together with the corresponding organism, accession number, peptides, theoretical isoelectric point (pI), molecular weight (Mw), and annotation of signal peptides are listed. Molecular weight and pI were not available for identified EST-clones. When, database searches lead to the identification of the same protein for several spots data were summarized. Individual databases are indicated as a) TREMBL, b) TIGR and c) Swissprot. Annotations for signal peptide were estimated from N-terminal sequences (70 aa) with SignalP 3.0 (http://www.cbs.dtu.dk/services/SignalP) and the following settings: organism was eukaryotes and method was hidden Markov model with a positive result at a probability higher than 0.55).

**Spot**	**Description**	**Organism**	**Accession**	**Peptides**	**mW**	**pI**	**Signal**
*Hydrolyzing O-glycoside*							
1, 2, 3, and 4	Xylan 1,4 beta xylosidase ^a^	*P. tremula*	Q2MCJ5	(K)AVSNNFATLMR(L)	81423	6,0	yes
	LEXYL2 protein Fragment ^a^	*S. lycopersicum*	Q76MS4	(K)VTQQDLDDTFNPPFK(S)	68868	7,7	no
				(K)VDMTNMNMR(A)			
	homologue to LEXYL2 protein Fragment Q76MS4 ^b^	*S. tuberosum*	TC145815	(T)AVSNNFATLMR(T)			no
				(T)VTQQDLDDTFNPPFK(C)			
				(T)AVSNNFATLMR(T) Ox (M10)			
	similar to Q76MS4 LEXYL2 protein Fragment partial 37 ^b^	*N. tabacum*	TC13056	(A)LPMTWYPQSYADK(T)			
				(A)LPMTWYPQSYADK(T) Ox (M3)			
				(G)VDMTNMNMR(G)			
				(A)GPTVFNFGDGLSYSNYK(C)			
12, 14, 15, 22	Xylan 1,4 beta xylosidase ^a^	*P. tremula*	Q2MCJ5	(K)AVSNNFATLMR(L)	81423	6,0	Yes
18, 20, 21,	Beta xylosidase like protein ^a^	*A. thaliana*	Q9LXD6	(R)LGFFDGDPK(S)	83168	7,2	yes
22, and 23	LEXYL2 protein Fragment ^a^	*S. lycopersicum*	Q76MS4	(K)VTQQDLDDTFNPPFK(S)	68868	7,7	no
	homologue to Q76MS4 LEXYL2 protein Fragment partial 88 ^b^	*S. tuberosum*	TC145815	(C)AVSNNFATLMR(G)			no
				(T)VTQQDLDDTFNPPFK(C)			
	similar to Q76MS4 LEXYL2 protein Fragment partial 37 ^b^	*N. tabacum*	TC13056	(C)LPMTWYPQSYADK(T)			
25	Alpha galactosidase Fragment ^a^	*Petunia hybrida*	Q6UAY5	(K)TFASWGVDYLK(Y)	31305	4,7	no
27	LEXYL2 protein Fragment ^a^	*S. lycopersicum*	Q76MS4	(K)VTQQDLDDTFNPPFK(S)	68868	7,7	no
	Xylan 1,4 beta xylosidase ^a^	*P. tremula*	Q2MCJ5	(K)AVSNNFATLMR(L)	81423	6,0	yes
	homologue to Q76MS4 LEXYL2 protein Fragment partial 88 ^b^	*S. tuberosum*	TC145815	(T)VTQQDLDDTFNPPFK(C)			no
				(T)AVSNNFATLMR(T)			
34	Glucan endo 1,3 beta glucosidase acidic isoform PR Q precursor ^c^	*N. tabacum*	P36401	(R)IYDPDQPTLEALR(G)	36971	5,0	yes
				(R)YIAVGNEVSPLNENSK(Y)			
				(K)YVPVLLNAMR(N)			
				(R)NIQTAISGAGLGNQIK(V)			
				(K)VSTAIETGLTTDTSPPSNGR(F)			
35	Alpha galactosidase ^a^	*C. canephora*	Q5DUH7	(K)LGIYSDAGTQTCSK(T)	41203	5,5	no
37, 38	Xyloglucan endotransglucosylase hydrolase XTH3 ^a^	*S. lycopersicum*	Q6RHY0	(R)IIFYVDGTPIR(E)	32134	5,1	yes
	homologue to Q6RHY0 Xyloglucan endotransglucosylase ^b^ hydrolase XTH3 partial	*N. tabacum*	TC5192	(A)IIFYVDGTPIR(T)			yes
				(C)APFSASYR(A)			
	homologue to Q6RHY0 endotransglucosylase hydrolase XTH3 ^b^ partial	*S. lycopersicum*	TC186880	(G)NSESIGVSYPK(T)			yes
41	LEXYL2 protein Fragment ^a^	*S. lycopersicum*	Q76MS4	(K)VTQQDLDDTFNPPFK(S)	68868	7,7	no
	homologue to Q76MS4 LEXYL2 protein Fragment partial 88 ^b^	*S. tuberosum*	TC145815	(T)VTQQDLDDTFNPPFK(C)			no
				(T)AVSNNFATLMR(T)			
52	LEXYL2 protein Fragment complete ^b^	*S. lycopersicum*	TC170394	(T)ISMLVNTAGSVSR(T)			no
57	Glucan endo 1 3 beta glucosidase acidic isoform PR Q ^c^	*N. tabacum*	P36401	(R)IYDPDQPTLEALR(G)	36971	5,0	yes
*Protease acitivity*							
57	Subtilisin like protease ^a^	*N. tabacum*	A9XG40	(R)TPSFLGLDR(S)	81136	6,6	yes
				(K)GYETTLGPVDVSK(E)			
	Subtilisin like protease ^a^	*N. tabacum*	A9XG41	(R)TPSFLGLDR(S)			
				(K)GYETTLGPVDVSK(E)	81123	6,7	yes
6	Subtilisin like protease ^a^	*N. tabacum*	A9XG40	(K)GYETTLGPVDVSK(E)	81136	6,6	yes
				(K)VSTVFSPSNSVK(V)			
				(K)VSVEPETLVFTR(A)			
	Subtilisin like protease ^a^	*N. tabacum*	A9XG41	(K)GYETTLGPVDVSK(E)	81123	6,7	yes
				(K)VSTVFSSSNSVK(V)			
				(K)VSVEPETLVFTR(V)			
*Chitinases activity*							
45, 46,	CHIP TOBAC Acidic endochitinase P precursor ^c^	*N. tabacum*	P17513	(R)GPIQLTNQNNYEK(A)	27452	4,7	yes
				(R)QDLVNNPDLVATDATISFK(T)			
47	Chitinase ^a^	*M. charantia*	Q207U1	(R)SFPGFGTTGDDATR(K)	34034	8,1	yes
	CHIQ TOBAC Acidic endochitinase Q precursor ^c^	*N. tabacum*	P17514	(R)WTPSAADQAANR(V)	27615	4,9	yes
	CHIP TOBAC Acidic endochitinase P precursor ^c^	*N. tabacum*	P17513	(R)GPIQLTNQNNYEK(A)	27452	4,7	yes
*Peroxidase activity*							
11	Peroxidase ^a^	*P. sativum*	Q18PR1	(R)ISPLTGTNGEIR(K)	34257	8,2	yes
	Peoxidase like protein ^a^	*A. thaliana*	Q0WLG9	(K)DAAPNANSAR(G)	38084	5,6	yes
	similar to Q9LEH3 Peroxidase precursor partial 91 ^b^	*N. tabacum*	TC12826	(T)GFDVVDNIK(A)			yes
24	Lignin forming anionic peroxidase ^c^	*N. tabacum*	P11965	(K)DAPANVGAGGFDIVDDIK(T)	34652	4,5	yes
				(K)GPSWQVLFGR(K)			
				(K)GMDLTDLVALSGAHTFGR(A) Ox (M2)			
				(K)LGNISPLTGTNGQIR(T)			
*Protein binding and Proteinase inhibitor activity*							
36	Biotic cell death associated protein ^a^	*N. glutinosa*	Q850R9	(K)IEDGFVTTGGIK(G)	25141	8,6	yes
49	Biotic cell death associated protein ^a^	*N. glutinosa*	Q850R9	(K)IEDGFVTTGGIK(G)	25141	8,6	yes
*Pathogenesis related protein family 1 – unknown function*							
55, 58	Pathogenesis related protein 1B ^c^	*N. tabacum*	P07053	(K)AVEMWVDEK(Q)	18487	5,2	yes
				(K)AVEMWVDEK(Q) Ox (M4)			
	Pathogenesis related protein 1A ^c^	*N. tabacum*	P08299	(K)AVEMWVDEK(Q)	18561	4,6	yes
				(K)AVEMWVDEK(Q) Ox (M4)			
	Pathogenesis related protein 1C ^c^	*N. tabacum*	P09042	(K)AVEMWVNEK(Q)	18570	5,2	yes
