# Supplementary figures and images for: Protein and Metabolite Analysis Reveals Permanent Induction of Stress Defense and Cell Regeneration Processes in a Tobacco Cell Suspension Culture
Source: Int J Mol Sci. 2009 Jul 6;10(7):3012–32. doi: 10.3390/ijms10073012 (PMC2738909; doi:10.3390/ijms10073012)

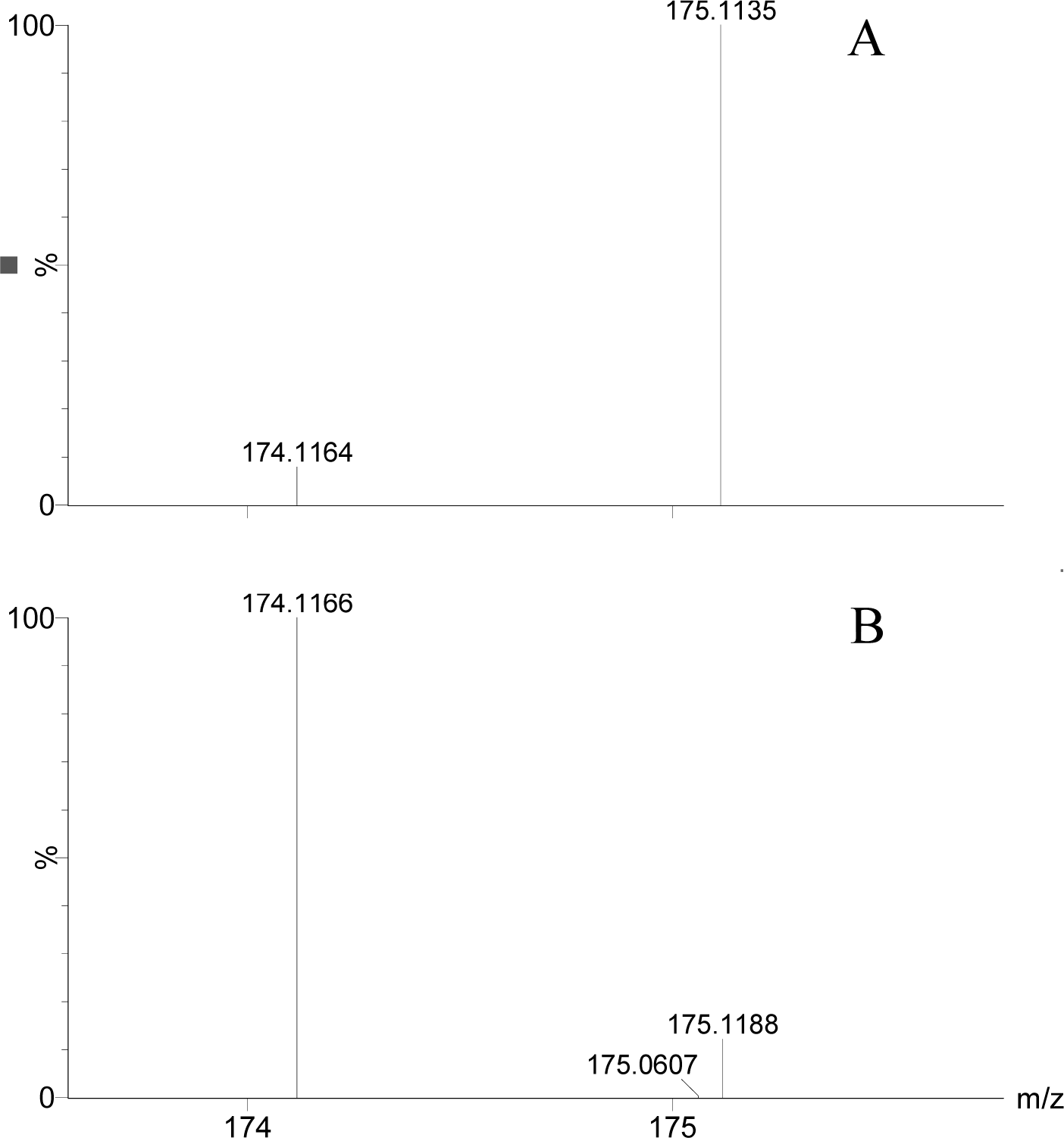

Supplement: Figure S1. — A) The isotope shift after 14d of cultivation with 15N labelled culture medium. B) Natural isotope pattern of the putrescine fragment. [file ijms-10-03012-s001.tif]

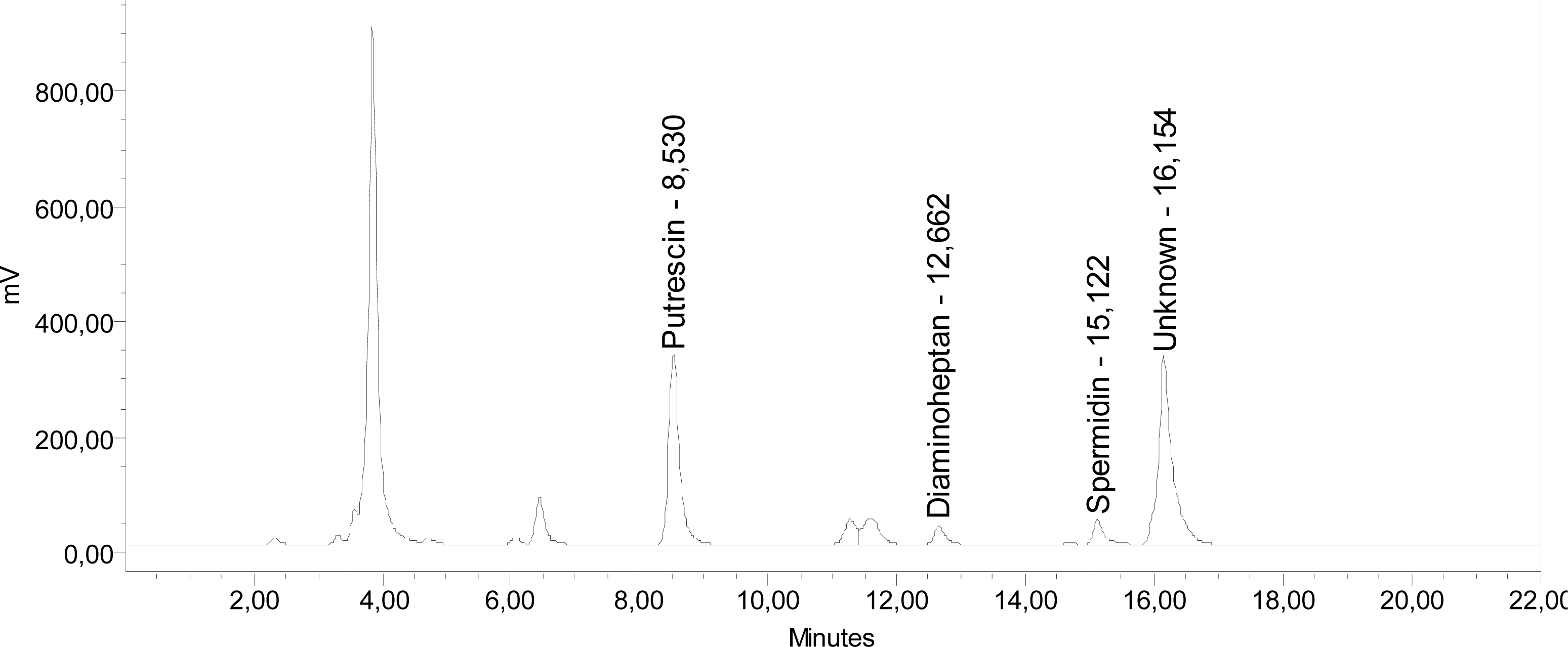

Supplement: Figure S2. — Polyamines were measured by fluorescence detection at 340 nm (excitation) and 510 nm (emission). The identification of polyamines was performed with authentic standards. [file ijms-10-03012-s002.tif]
